# Design, reporting, and research focus of neurology and psychiatry drug trials (2020–2023): a registry-based meta-epidemiological study using ClinicalTrials.gov and NLP

**DOI:** 10.1186/s13063-026-09909-8

**Published:** 2026-07-28

**Authors:** Simona Emilova Doneva, Beate Sick, Tilia R. Ellendorff, Jean-Philippe Goldman, Daniel S. Reich, Lars G. Hemkens, Gerold Schneider, Benjamin Victor Ineichen

**Affiliations:** 1 Center for Reproducible Science and Research Synthesis, University of Zurich, Zurich, Switzerland; 2 TIDIT Thurgau Institute for Digital Transformation at the University of Konstanz and HTWG Konstanz, Kreuzlingen, Switzerland; 3 Department of Computational Linguistics, University of Zurich, Zurich, Switzerland; 4 National Institute of Neurological Disorders and Stroke, National Institutes of Health, Bethesda, MD USA; 5 Department of Clinical Research, University of Bern, Bern, Switzerland

**Keywords:** CNS drug trials, Psychiatry, Neurology, ClinicalTrials.gov, Alzheimer’s disease, Parkinson’s disease, Schizophrenia, Depression, Multiple sclerosis, Pain, Clinical trial registries, Natural language processing (NLP), Trial design, Results reporting

## Abstract

**Background:**

Clinical trial registries document trial design, funding, and results reporting, but heterogeneous and unstructured entries limit cross-trial comparison. Consequently, it is unclear how neurology and psychiatry drug trials are designed and reported in practice, hampering comparison of studies, understanding of the available evidence, and planning of future trials. The aim of this study was to quantify trial design features, results reporting practices, and research focus in neurology and psychiatry drug trials using harmonized registry data.

**Methods:**

This is a registry-based observational study of interventional neurology and psychiatry drug trials registered on ClinicalTrials.gov between 2000 and 2023. Using natural language processing (NLP) with manual validation, we extracted and harmonized trial information on phase, randomization, masking, sample size, funding source, results reporting, and target diseases and drugs. We also performed content-based clustering and visualization to explore patterns across trials by drug and disease category. We analyzed temporal trends and differences across trial characteristics.

**Results:**

We included 18,609 trials. Most were early phase, single-center, and small, typically enrolling 11–50 participants; 60% were completed. Industry funded 36% of trials, with a declining share over time and a corresponding increase in university sponsorship. Ten percent of trials were non-randomized and 38% were open label. Among trials completed after 2007, 50% reported results, with a mean delay of 12 months. The most frequently studied conditions were pain (10%), schizophrenia (8%), depression (8%), and Alzheimer’s disease (7%). Drug testing was widely distributed, with atypical antipsychotics, levodopa, and ketamine each accounting for 1–2% of trials. Content-based clustering identified disease-specific groupings of trials. Harmonized data are available through an interactive dashboard.

**Conclusions:**

Registered neurology and psychiatry drug trials show persistent limitations in design and results reporting despite two decades of registry requirements. Registry-based analyses using computational harmonization can support empirical evaluation of trial practices and inform future trial design and reporting.

**Supplementary Information:**

The online version contains supplementary material available at https://doi.org/10.1186/s13063-026-09909-8.

## Introduction

Neurological and psychiatric diseases (hereafter, neuropsychiatric diseases) affect millions of people worldwide, imposing a high burden on individuals and health systems [1]. Despite this, many remain difficult to treat effectively, and drug development in these fields is often slow and failure is common [2]. This has led to sustained clinical research activity, with numerous drug trials registered and conducted each year.

ClinicalTrials.gov is a primary global registry of clinical trials. Since the Food and Drug Administration Amendments Act of 2007, registration and results submission have been mandated for many interventional trials of U.S. Food and Drug Administration (FDA)-regulated drugs, biologics, and devices. However, its usefulness for empirical analysis of trial practice is limited because many key fields, including target diseases and drugs, are recorded as free text. This free-text input leads to inconsistent labeling and a lack of standardization, with substantial variation in terminology (e.g., “stroke” vs “cerebrovascular accident” for the same condition) [3–5]. Despite platforms such as the Aggregate Analysis of ClinicalTrials.gov (AACT) improving access to structured data, free-text entries remain largely unstandardized and resist automated validation and harmonization [6, 7]. Manual synthesis of this volume of registry data has been feasible only in narrow subfields and is not feasible at scale [8].

This limits empirical comparison of trials, assessment of reporting practices, and understanding of which drugs and diseases are most commonly studied, and where gaps for future clinical research may lie [9, 10].

Advances in natural language processing (NLP), a set of computational methods for extracting and structuring information from unstructured text, enable harmonization of registry data at scale [11, 12]. These tools may help address the challenges outlined above. In this study, we combined NLP-derived disease and drug information with structured AACT data to enable cross-trial comparison of trial design and reporting practices. Our objectives were to (1) extract and harmonize disease and drug information from free-text registry fields using NLP; (2) quantify trial design characteristics and sponsorship patterns; (3) assess trends in trial rigor, including randomization and masking, and results reporting, with subgroup comparisons of phase 2 versus phase 3 trials; and (4) examine the distribution of target diseases and drugs over time. In addition, we provide a curated and harmonized dataset, accessible freely online to facilitate secondary research that can inform and improve future trial planning.

## Methods

### Study design and outcomes

Retrospective bibliometric analysis of ClinicalTrials.gov (2000–2023) using natural language processing (NLP). The analysis focused on interventional drug trials related to neurological and/or psychiatric diseases. The outcomes of interest were the annual number of registered neuropsychiatric drug trials; the distribution of trial characteristics, including phase, design features, geographical location, number of study countries, number of study sites, enrollment size, and outcome structure; the proportion of trials by sponsorship category; the proportion of completed trials with results disclosed in ClinicalTrials.gov and the time to results reporting; the proportion of trials reporting key methodological features, including randomization and masking; and the relative frequency and evolution over time of target diseases/conditions and drugs investigated. Subgroup analyses compared these outcomes between phase 2 and phase 3 trials. Reporting followed the Reporting of Studies Conducted Using Observational Routinely Collected Health Data (RECORD) statement [13].

### Data collection

We used the latest available version of the AACT database (accessed March 07, 2024). Along with the study identifier (NCTID), official title, and brief summary description, we extracted the following trial characteristics from AACT: (1) start date, (2) completion date, (3) phase, (4) overall status, (5) condition name, (6) intervention name, (7) intervention type, (8) country or countries where the trial was conducted, (9) primary outcomes, (10) allocation, (11) masking, (12) intervention group type as recorded in the trial design groups, (13) number of participants enrolled, and (14) sponsorship. In addition, we used pre-calculated summary fields provided in the AACT calculated values table for the (15) number of study sites, defined as the number of listed study facilities; (16) results reporting status; (17) time from trial completion to results reporting; (18) number of primary outcomes measured; (19) number of secondary outcomes measured; and (20) number of other outcomes measured. Time to results reporting is defined as the number of full months between the study’s primary completion date and the date on which results were first submitted to ClinicalTrials.gov [14]. Further details on extracted variables and their source tables are provided in Supplementary Table 1.

### Data selection and preprocessing

#### In- and exclusion criteria

We included trials if they (1) were registered on ClinicalTrials.gov between 2000 (the registry’s launch year) and 2023; (2) were classified as interventional studies; (3) tested at least one drug intervention; and (4) targeted a neurological or psychiatric disease (neuropsychiatric).

Neuropsychiatric diseases were identified using a predefined list of conditions derived from ICD-11 and MeSH, two widely used medical classification systems (18,831 conditions including synonyms in total). This list and the associated in- and exclusion criteria were developed and validated in our prior study, which also served as the benchmark for training and validating the NLP pipeline used in the present analysis [15]. Further details are provided in Supplementary Sect. 13.

We excluded trials if they did not involve drug interventions, were not interventional in design, or did not have a condition that exactly matched an entry in the predefined neuropsychiatric disease list..

Sponsors were grouped into industry, university, hospital, governmental, or other (see Supplementary Tables 2–4).

### Identification of drugs and diseases in trials

#### Named entity recognition

In the AACT database, the disease and intervention fields are entered as free text by trial registrants, with some additional matching to MeSH terms by the AACT team. This can lead to inconsistencies because the free-text entries are often unstandardized or use varying terminology, and the rule-based matching may miss newer or less common terms. To mitigate these limitations, we combined disease and drug information from the structured AACT fields with entities extracted from unstructured registry text using machine learning–based named entity recognition (NER) [16–18].

In our previous study, we created and validated a dataset of 1093 manually annotated ClinicalTrials.gov entries (trial title and brief summary), focusing on disease and drug names [15]. BioLinkBERT, the best-performing language model from that work, was applied here to extract disease and drug mentions from trial titles and brief summaries using NER [19]. To simplify analysis, we kept only unique terms for each trial. We expanded abbreviations to their full forms using the Schwartz-Hearst algorithm (e.g., “MS” to “multiple sclerosis”) [20]. The final disease and intervention sets therefore comprised the union of AACT-derived and NER-derived entities.

#### Named entity linking

Clinical trial registries often use different terms to describe the same concept, such as “stroke” and “cerebrovascular accident.” To standardize these variations, we applied named entity linking to align each term with the most appropriate match from a medical ontology [21].

First, we used a dictionary-based method to identify potential matches [22]. The target list of drug names was a compilation of the DrugBank, Wikipedia, Medline Plus, and MeSH terms (https://pypi.org/project/drug-named-entity-recognition/). For diseases, we reused the list of neuropsychiatric diseases compiled for filtering the database.

Second, we used the Systematized Nomenclature of Medicine Clinical Terms (SNOMED CT), regarded as one of the most comprehensive healthcare terminologies [23]. SNOMED allowed us to more easily group related entities to a higher level category, e.g., “progressive MS” and “relapsing MS” to their common parent “MS.” We used SapBERT, a model for biomedical concept mapping and representation, to link extracted entities to the ontology [24].

### 2D atlas to map the ClinicalTrials.gov database

To explore and visualize thematic patterns among trials, we created a two-dimensional map of the dataset. Trial titles and summaries were first converted into numerical representations that capture their meaning using the PubMedBERT language model. These high-dimensional data were then projected to two dimensions with t-SNE [25, 26]. Each point on the resulting 2D map represents a single trial, with colors indicating associated diseases or drugs [27]. Trials were labeled as “Unlabeled” when none of the disease- or drug-related keywords in the predefined coloring dictionary matched the trial record. An interactive version of this atlas is available through the NeuroTrialomics Dashboard (https://sed-stride-lab.shinyapps.io/neurotrialomics-dashboard1/).

### Statistical and trend analysis

We examined temporal trends in clinical trial characteristics, including funding sources, randomization, masking, and results reporting. To model non-linear trends over time, we used generalized additive models (GAMs) with a binomial family. In addition, we used Spearman’s rank correlation to describe the strength and direction of these trends. For disease and drug categories, we calculated the yearly number of unique trials (adjusted to proportions) and visualized temporal patterns with heatmaps (including diseases with > 100 mentions and drugs with > 60 mentions). Differences in proportions between groups (e.g., trial phases) were tested with chi-square tests. Descriptive statistics were computed for key trial features.

Statistical significance was set at α = 0.05 (two-tailed). All analyses were performed in R (version 4.2.2), with descriptive analyses and visualizations in Python (version 3.11.7). The full source code and processed data are available at https://github.com/Ineichen-Group/NeuroTrialAnalysis.

## Results

### Included clinical trials

From 485,756 trials retrieved from ClinicalTrials.gov on March 07, 2024, via AACT interface, 60,259 (12%) were related to neurological and/or psychiatric (neuropsychiatric) diseases. Of these, 46,376 (10%) were interventional, and our final analysis included 18,609 (4%) drug intervention trials (Supplementary Fig. 1). Most of these trials related to treatment (77%, Supplementary Fig. 2 A) and most were completed (61%, Supplementary Fig. 2B).

**Fig. 1:**
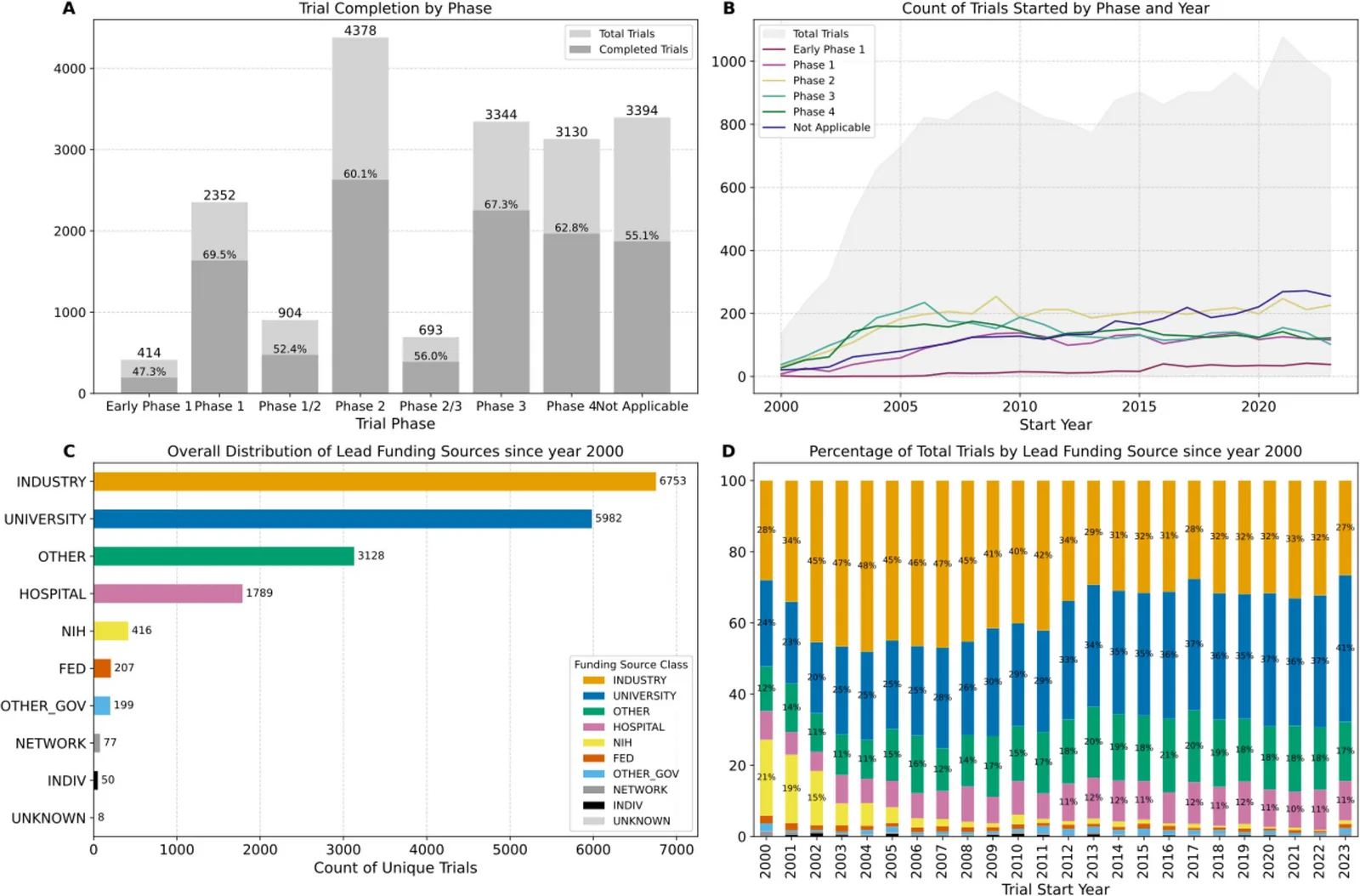
Overview of clinical trial phases and funding sources. **A** Total number of trials across different phases and the proportion of trials completed within each phase. **B** Count of trials started annually, categorized by phase, showing trends in trial initiation over time. **C** Overall distribution of lead funding sources for trials. **D** The yearly percentage distribution of trials by lead funding sources, reflecting the shifts in funding dynamics across time

Trial registrations increased substantially over time, from 136 newly registered neuropsychiatric drug trials in 2000 to 948 in 2023, with annual counts generally stabilizing at approximately 800–1000 trials after 2006 (Fig. 1B).

### Geographical distribution and trial phases

The United States accounts for nearly half of all registered trials (49%), followed by Canada (9%) and several European countries (notably France, Germany, and the UK, Supplementary Fig. 3). Most trials were conducted in a single country (86%) and 8% of trials did not report a country.

Phase 2 is the single largest category (24%), followed by phase 3 (18%) and phase 4 (17%). 18% of trials had no formal phase designation (Fig. 1A and B). According to ClinicalTrials.gov documentation, the N/A (not applicable) designation is used when conventional drug-development phases do not apply, for example in some device or behavioral studies. We manually reviewed 20 randomly sampled phase not applicable trials, categorizing study type and assessing whether the designation was appropriate. Nutritional interventions were most common (25%), while treatment-optimization, device or imaging, and investigational therapeutic studies each accounted for 20%. The designation was justified or likely justified in 65%, uncertain in 15%, and likely inappropriate in 20%, mainly for investigational or efficacy-focused drug studies (Supplementary Tables 5 and 6).

### Trial sponsorship

Overall, industry was the largest sponsor, funding 36% of trials. Its share declined over time, from 45% in 2002 to 27% in 2023 (*p* < 0.001). In contrast, university sponsorship doubled from 20 to 41% (*p* < 0.001), while NIH sponsorship decreased steadily (*p* < 0.001) (Fig. 1C and D).

Industry was most active in phases 1–3 (60–79%), whereas universities were more active in early phase 1 (89%) and phase 2/3 (60%). Universities also sponsored most phase 4 (64%) and unassigned-phase trials (89%) (Supplementary Figs. 4 A, 4B, and 5 A). Over time, industry-funded phase 2 and 3 trials declined, while university-funded phase 2 trials increased, increasingly narrowing the gap between the two (Supplementary Fig. 5B).

### Trial design

**Fig. 2:**
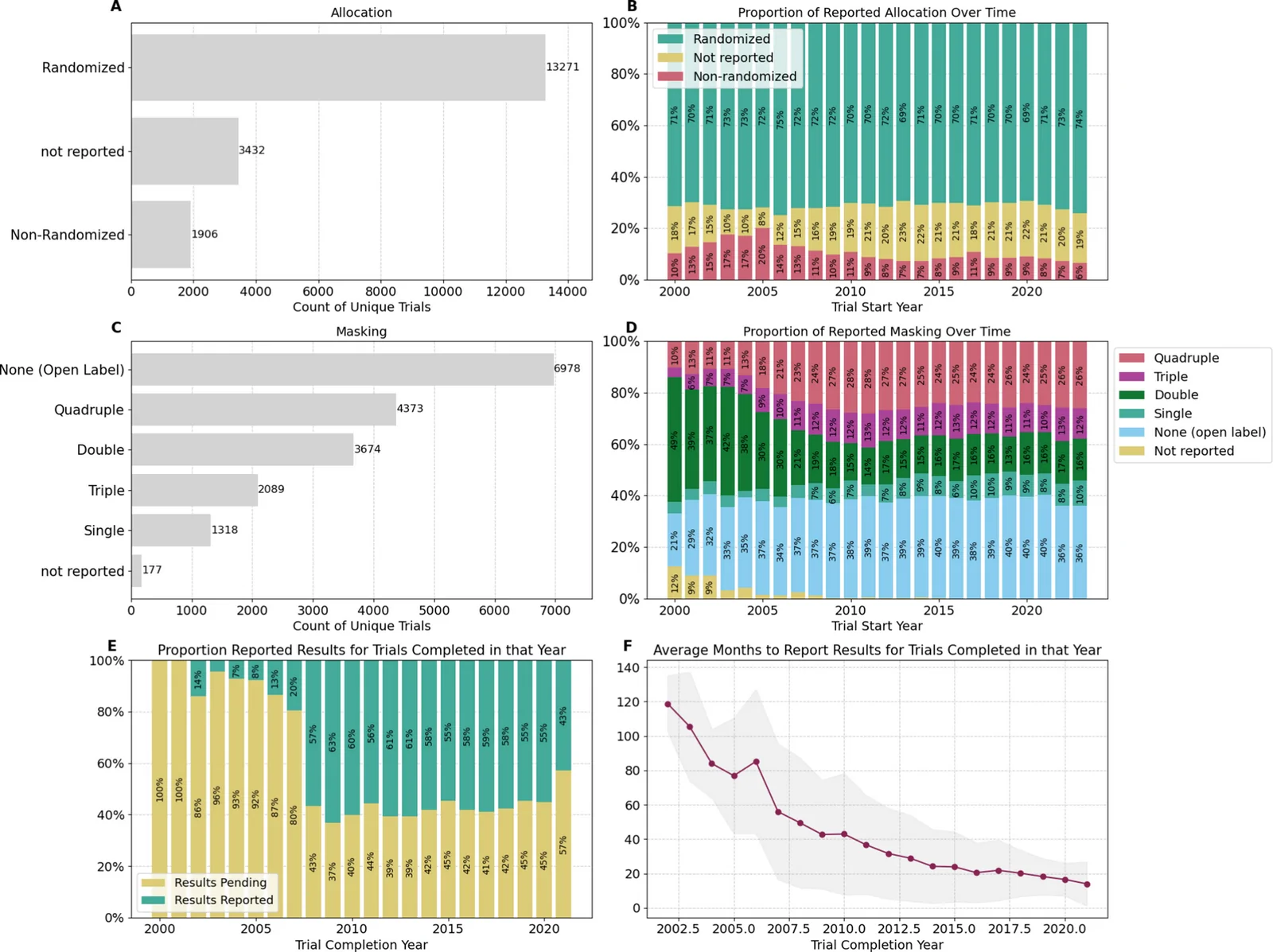
Allocation, masking, and results reporting trends. **A** Frequency distribution of trials by allocation method. **B** Proportion of reported allocation methods over time. **C** Frequency distribution of masking types used in trials. **D** Proportion of reported masking methods over time. **E** Proportion of trials that have reported results versus those still pending for each year of trial completion. **F** Average time (in months) taken to report results for completed trials, along with the standard deviation. For panels **E** and **F**, early phase 1, phase 1, and not applicable trials were excluded, and only trials with completion year < 2022 were included to account for expected reporting delays

Most trials (71%) used random allocation, while 10% were non-randomized and 18% did not report their allocation method (Fig. 2A). The overall proportions were stable over time, though non-randomized designs became slightly less common after 2005 (*p* < 0.001) (Fig. 2B). Missing information on randomization was more frequent in phase 2 than in phase 3 (18% vs 14%, *p* < 0.001) (Supplementary Fig. 5 C).

Most trials (61%) used some form of masking, while 38% were conducted open label and 1% did not report masking status (Fig. 2C). The use of double masking declined after 2000 (*p* < 0.001) (Fig. 2D). Masking patterns differed across comparator categories, with placebo-comparator trials more commonly using masked designs, whereas trials with no comparator information included a large proportion of open-label designs (Supplementary Fig. 6). Among masked trials, 12% did not specify which trial roles were masked. Double-masked trials most commonly masked participants and investigators, while triple-masked trials most often masked participants, care providers, and investigators or outcome assessors. Single-masked trials most often blinded outcome assessors or participants (Supplementary Fig. 7). Phase 3 trials were slightly more likely than phase 2 to use double masking (24% vs 20%, *p* < 0.001) (Supplementary Fig. 5D).

Most trials measured a single primary outcome (68%), and 27% reported 2–5 secondary outcomes. Single-site studies were most frequent (57%). Trial size was typically modest, with 36% enrolling 11–50 participants and 30% enrolling 100–1000 (Supplementary Fig. 8A–D).

### Trial results reporting

For this analysis, early phase 1, phase 1, and not applicable trials were excluded because results-reporting requirements differ and are generally less stringent for these categories. Before the introduction of mandatory results reporting in 2007, about 80% of completed trials did not report results within the registry (Fig. 2E). Reporting improved thereafter, with roughly 60% of completed trials since 2007 disclosing outcomes (*p* < 0.001) (Fig. 2F). The mean delay to reporting has decreased over time but remains about 12 months after trial completion.

Phase 3 trials disclosed results slightly more often than phase 2 (51% vs 50%, *p* < 0.001). Among trials completed after 2008, reporting rates reached about 70% (Supplementary Fig. 5E).

### Prevalent target diseases/conditions

**Fig. 3:**
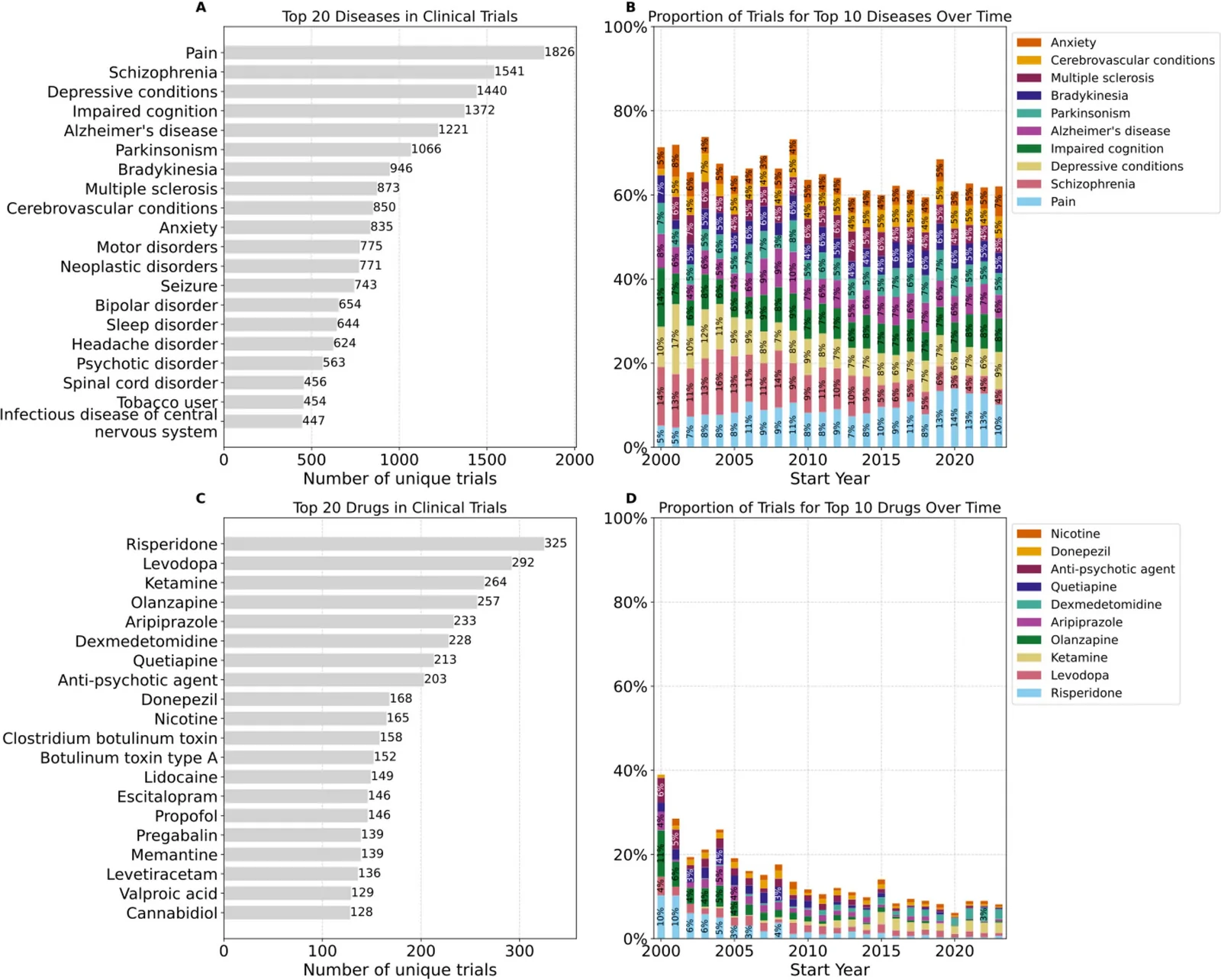
Summary of the most prevalent diseases and drugs. **A** The 20 most frequent diseases mentioned in the clinical trials. **B** The proportion of clinical trials over time for the top 10 diseases. **C** The top 20 most common drugs mentioned in the clinical trials. **D** The proportion of clinical trials over time for the top 10 drug interventions

From 9,509 distinct disease terms, pain was the most frequently studied condition (10%), followed by schizophrenia and depression (each 8%). Impaired cognition and Alzheimer’s disease accounted for 7% each, Parkinsonism for 6%, and multiple sclerosis, cerebrovascular disorders, and anxiety for 4–5% each (Fig. 3A). Phase 2 studies focused mainly on impaired cognition and Alzheimer’s disease, while phase 3 focused on pain and schizophrenia (Supplementary Figs. 9 A and B).

**Fig. 4:**
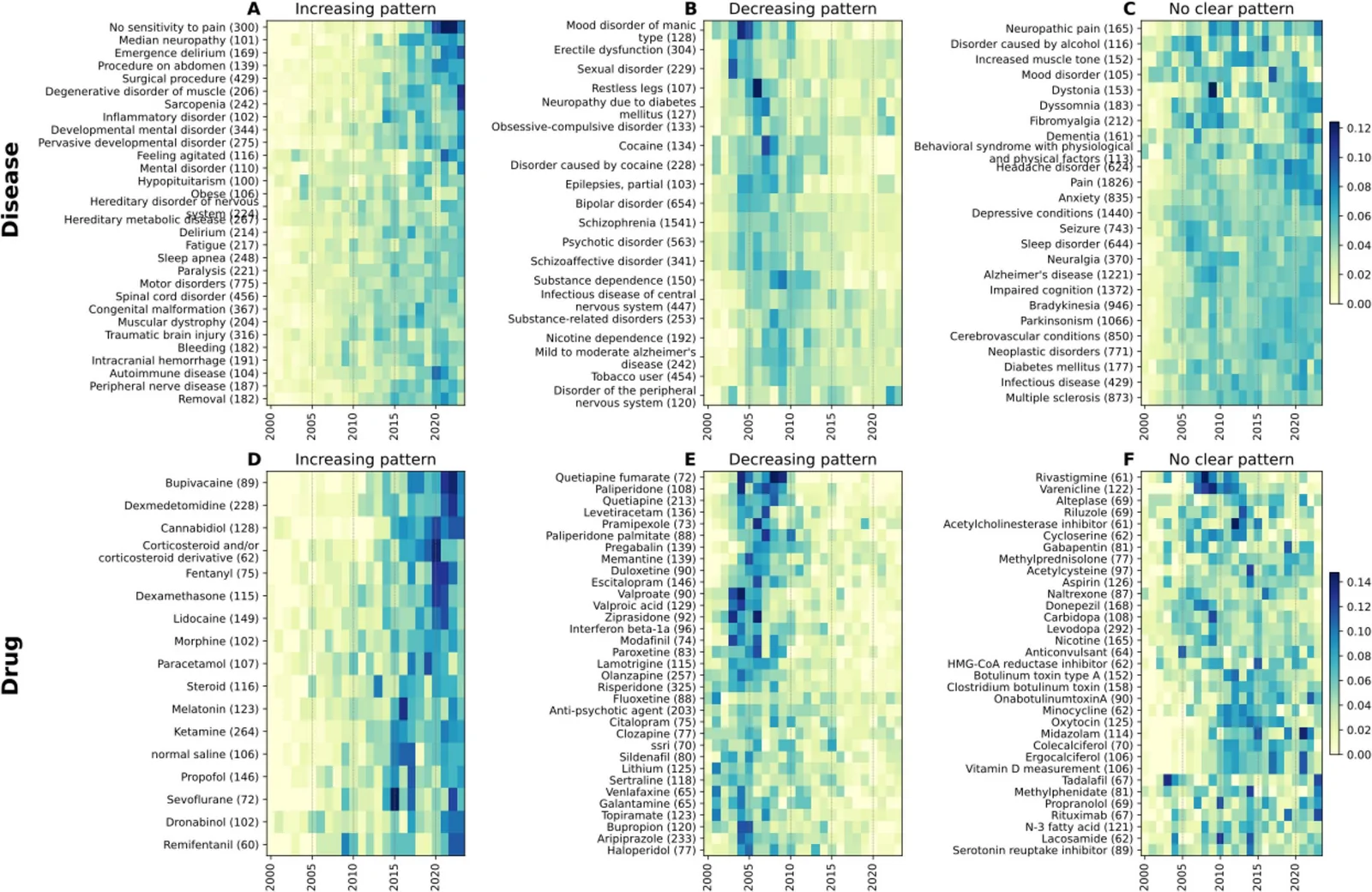
Disease and drug trends of trials conducted over the period 2000–2023. Panels **A**, **B**, and **C** (top row) display medical conditions with more than 100 trials, while panels **D**, **E**, and **F** (bottom row) show drugs with more than 60 trials. The y-axis lists diseases or drugs with total trial counts in parentheses, and the x-axis represents the study years. Darker colors indicate a higher proportion of trials started in that year. For both diseases and drugs, the data are divided into three sections based on observed trends: increasing trend (**A**, **D**), decreasing trend (**B**, **E**), and unclear or stable trend (**C**, **F**)

Over time, trial activity increased for musculoskeletal and cerebrovascular diseases, as well as traumatic brain and spinal cord injury, while trials on psychiatric conditions (including schizophrenia/psychosis, substance use disorders, and sexual disorders) declined (Figs. 3B and 4A–C). In contrast, several major conditions such as Alzheimer’s disease, Parkinsonism, and multiple sclerosis showed stable trial activity without clear upward or downward trends.

### Prevalent target drugs

Among the 16,282 unique drug names identified, a wide range of agents have been studied, with no single drug dominating the landscape. The most frequently mentioned were atypical antipsychotics such as risperidone, olanzapine, aripiprazole, and quetiapine (each 1–2%), alongside levodopa (2%), ketamine (1%), and dexmedetomidine (1%) (Fig. 3C).

Over time, trials increasingly focused on anesthetic and pain-management drugs (e.g., fentanyl, morphine, ketamine), as well as newer therapies including cannabinoids and sleep-regulating substances, most often in phase 2 studies (Supplementary Fig. 9 C and D). In contrast, antipsychotics (e.g., quetiapine), antidepressants (e.g., escitalopram), and cognitive enhancers such as modafinil declined in prevalence (Figs. 3D and 4D–F).

### 2D Map of clinical trials

**Fig. 5:**
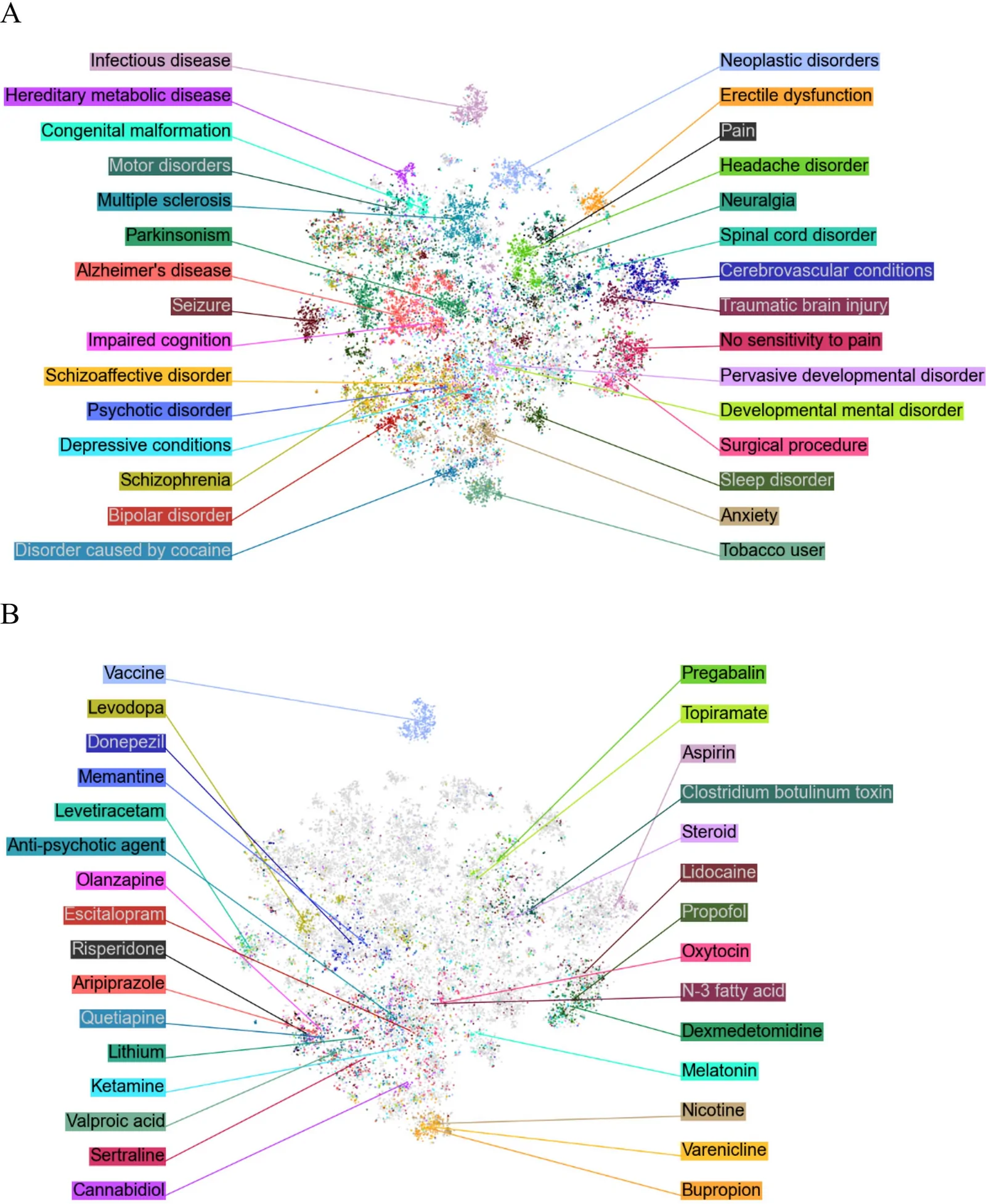
2D embedding of the clinical trials dataset. Trial brief summaries (*n* = 18.6 K) were transformed into 768-dimensional vectors with PubMedBERT and then embedded in 2D with t-SNE. Colored after clustering using keywords for 30 most frequent disease (**A**) and drug names (**B**). The labels are positioned in the areas of highest density of trials with this keyword. Trials not assigned to one of the displayed keyword groups (unlabeled) are shown in gray and plotted in the background (for A = 3.4 K, for B = 13.9 K)

When trials are grouped by similarities in their content, patterns of related topics become visible. The 2D map shows how clinical trials cluster based on the meaning and context of their titles and summaries (Fig. 5A and B, or as interactive dashboard: https://sed-stride-lab.shinyapps.io/neurotrialomics-dashboard1/). Trials clustered by disease into distinct groups covering infections and vaccines, congenital and metabolic disorders, multiple sclerosis, Alzheimer’s and Parkinson’s disease, pain conditions, cerebrovascular and traumatic disorders, and psychiatric diseases (Fig. 5A; see Supplementary Figs. 10–11 and Supplementary Results for details).

## Discussion

### Summary of findings

The objective of this study was to use NLP to extract and harmonize data from clinical trial registries, enabling a large-scale overview of drug trials in neurological and psychiatric diseases. We found that the typical trial was small, single-center, randomized, and in phase 2, yet many studies still lacked or failed to report methodological rigor. Results reporting has improved in recent years but remains incomplete. Research activity was concentrated on a few major conditions, particularly pain, schizophrenia, depression, Alzheimer’s disease, and Parkinsonism. A broad spectrum of drugs was tested, most often atypical antipsychotics, levodopa, and ketamine, with growing attention to anesthetic and pain-management agents and newer therapies such as cannabinoids.

### Trial design characteristics and results reporting

The proportion of non-industry, non-NIH sponsored studies has increased, particularly for early-stage clinical trials, as well as trials without an assigned phase, primarily sponsored by universities, hospitals, and nonprofit organizations. These findings are consistent with recent reports showing that approximately 60% of phase 2–4 neurology trials are industry-funded, but also that many pharmaceutical companies have either scaled back or discontinued their neuroscience programs [28, 29]. This shift has been attributed to higher failure rates, longer development times, and greater costs compared to drug development in other medical areas [2, 30, 31].

Although improving over time, a notable number of clinical trials did not employ randomization and/or masking. While further investigation is required to determine the total number of trials that do require such a design, it is likely that there is still room for improvement. The absence of these methods can lead to low internal validity and false-positive findings, which can result in high downstream costs and safety concerns for patients potentially exposed to ineffective or hazardous substances, likely also contributing to the low rates of regulatory approval in drug development [2, 32].

We observed an increase in trials reporting results and a reduction in reporting time for trials completed after 2007, likely due to the 2007 US law mandating timely results reporting for certain clinical trials. However, despite the regulations, about 40% of trials completed after 2007 still fail to publish results on ClinicalTrials.gov each year. This aligns with other studies, which have found low compliance with regulatory requirements for timely reporting of clinical trial results and raised concerns about the integrity of the evidence base for clinical medicine [28, 33]. However, it is likely that some clinical trials are reported within publications instead of trial registries [34].

### Trends in studied diseases and drugs

Pain-related conditions were the most frequently studied, including pain symptoms in disorders such as multiple sclerosis [35–38]. Schizophrenia was the second most common target but declined over time. Dementia-related disorders were also prominent, consistent with rising global disease burden [38, 39].

Most trials tested atypical antipsychotics targeting dopamine pathways [40, 41]. Trial numbers in schizophrenia declined, possibly reflecting shifts toward behavioral approaches and target discovery [41–45]. Ketamine emerged as a treatment for major depressive disorder, and semantic mapping revealed distinct therapeutic strategies in Parkinson’s disease [46–48].

### Limitations

The findings of this study should be interpreted with certain limitations in mind. First, while ClinicalTrials.gov captures a substantial portion of global clinical trials, it is not comprehensive. Some trials may be registered in other global registries, potentially leading to under-representation [49]. The World Health Organization’s International Clinical Trials Registry Platform (ICTRP) offers broader coverage, but its structure does not readily support large-scale data mining [50].

Second, we used automated entity recognition techniques to extract drug and disease information. Although these methods were validated in our prior study and showed high accuracy, the models can still produce occasional misclassifications or miss some entities [15].

Third, registry entries vary in completeness and use of free text, and even after harmonization, inconsistencies in reporting (e.g., trial phase, masking, or outcomes) may persist.

### Conclusions

This study demonstrates that NLP methods can enable empirical, cross-trial analyses of clinical trial registries that would not be feasible manually. Our approach provides a framework to extract and harmonize trial data, supporting comparison of trial design and reporting practices across diseases and trial characteristics in neuropsychiatry. Our analysis identified persistent limitations in results reporting and trial design, which reduce the interpretability and practical use of registered trial data. Addressing these limitations is important to improve the usability of trial registries for comparing studies and planning future trials. Beyond neuropsychiatry, our approach could be applied to other medical areas to support cross-trial analysis using registry data.

## Supplementary Information

Supplementary Material 1. Table S1–S6. Fig. S1–S11.

## Data Availability

All data and code that support the findings of this study are available in our public GitHub repository (https://github.com/Ineichen-Group/NeuroTrialAnalysis). For any questions regarding the data, metadata, or code analysis, contact the corresponding author, SED. The NeuroTrialomics Dashboard is available at https://sed-stride-lab.shinyapps.io/neurotrialomics-dashboard1/. To keep it up to date, we will regularly update the data using the ClinicalTrials.gov REST API. The dashboard is integrated into our GitHub repository for maintenance and version control.

## Abbreviations

AACT: Aggregate Analysis of ClinicalTrials.gov
AI: Artificial intelligence
FDA: Food and Drug Administration
GAM: Generalized additive model
ICD: International Classification of Diseases
ICTRP: International Clinical Trials Registry Platform
MeSH: Medical Subject Headings
NCTID: National Clinical Trial Identifier
NER: Named entity recognition
NIH: National Institutes of Health
NLP: Natural language processing
RECORD: REporting of studies Conducted using Observational Routinely-collected health Data
SNOMED CT: Systematized Nomenclature of Medicine; Clinical Terms
t-SNE: T-distributed stochastic neighbor embedding
