# Supplementary material for: Design, reporting, and research focus of neurology and psychiatry drug trials (2020–2023): a registry-based meta-epidemiological study using ClinicalTrials.gov and NLP
Source: Trials. 2026 Jul 28;27:622. doi: 10.1186/s13063-026-09909-8 (PMC13692975; doi:10.1186/s13063-026-09909-8)
Supplement: Supplementary file 1 [file 13063_2026_9909_MOESM1_ESM.docx]

**Supplementary results**

There are several distinct disease clusters (**Figure 5A**): (1) trials on infectious diseases and vaccines (top). (2) hereditary metabolic diseases and congenital malformations (left). (3) multiple sclerosis (central/top). (4) Alzheimer’s/Parkinson’s disease trials (middle-left). The left Parkinson’s cluster focuses on motor symptoms and levodopa-related topics, comprising mostly phase 2 and 3 trials. The right cluster represents newer trials addressing functional symptoms and novel therapies like deep brain stimulation, with a higher proportion of phase-unassigned studies (**Supplementary Figures 8A-D and 9A-D**). (5) Pain-related trials, including studies on headaches and neuralgia (middle-right). (6) Cerebrovascular and traumatic diseases (Far right). (7) psychiatric conditions, including schizophrenia, depression, bipolar disorder, and substance abuse disorders (bottom).

**Supplementary data**

## 1. Overview of included data

The analyzed dataset was compiled from multiple tables in the Aggregate Analysis of ClinicalTrials.gov database (AACT), which provides structured access to information submitted to ClinicalTrials.gov. Supplementary Table 1 lists the AACT source tables and variables used in the analysis, including study dates, phase, status, design characteristics, conditions, interventions, countries, outcome reporting, facility counts, and sponsor information.

Definitions of these ClinicalTrials.gov data elements are available at: https://clinicaltrials.gov/policy/protocol-definitions#intro.

**Supplementary Table 1**: Source table and columns used to compile the analyzed dataset for the current study.

| AACT Table | Columns |
| --- | --- |
| studies | brief_title, study_official_title, start_date, completion_date, phase, overall_status |
| brief_summaries | brief_summary_description |
| conditions | name |
| interventions | intervention_name, intervention_type |
| countries | name |
| designs | primary_purpose, allocation, masking, subject_masked, caregiver_masked, investigator_masked, outcomes_assessor_masked |
| design_groups | group_type |
| calculated_values | number_of_facilities, were_results_reported, months_to_report_results, number_of_primary_outcomes_to_measure, number_of_secondary_outcomes_to_measure, number_of_other_outcomes_to_measure |
| sponsors | agency_class, lead_or_collaborator |

## 2. Sponsor name re-assignment

As part of the data processing, we refined the classification of research sponsors by reassigning their agency class based on keyword matching in sponsor names. The lists of university- and hospital-related terms were developed by looking at examples from the records assigned as “OTHER” agency class category (Supplementary Table 2). This processing step affected 7,771 records, reclassifying 5,982 sponsors as universities and 1,789 as hospitals. Among those reclassified as universities, the original classifications were: OTHER (5,946), OTHER_GOV (28), FED (6), INDUSTRY (1), and UNKNOWN (1). For hospitals, the original classifications were: OTHER (1,570), OTHER_GOV (141), FED (77), and UNKNOWN (1).

**Supplementary Table 2**: Overview of keywords-based sponsor agency type assignment.

| Category | Assigned when sponsor name contains any of the following |
| --- | --- |
| UNIVERSITY | 'university', 'universita', 'université', 'universität', 'universiteit', 'universidad', 'universite', 'università', 'universiti', 'universitesi', 'universitário' |
| HOSPITAL  (if university does not apply) | 'hospital', 'hôpital', 'hopital', 'krankenhaus', 'klinikum', 'ospedale', 'ziekenhuis', 'sanatorio', 'medical center', 'health system', 'memorial', 'infirmary', 'med center', 'centro médico' |

To validate our reclassification, we manually reviewed 100 randomly sampled records from both the “UNIVERSITY” and “HOSPITAL” categories, achieving 100% accuracy. Around 20% of “UNIVERSITY” cases were university-affiliated hospitals or medical centers, highlighting institutional overlap. While our approach ensures high precision, we cannot assess recall due to unknown variations in university and hospital names in the database. Randomly sampled examples for the cases where re-classification affected the agency class label are given in Supplementary Table 3 and Supplementary Table 4.

**Supplementary Table 3**: Examples of changed agency class to "UNIVERSITY".

| NCTID | sponsor_name | agency_class | agency_class_refined |
| --- | --- | --- | --- |
| NCT00759317 | University of California, Los Angeles | OTHER | UNIVERSITY |
| NCT05475808 | Abant Izzet Baysal University | OTHER | UNIVERSITY |
| NCT05240976 | China Medical University Hospital | OTHER | UNIVERSITY |

**Supplementary Table 4**: Examples of changed agency class to "HOSPITAL".

| NCTID | sponsor_name | agency_class | agency_class_refined |
| --- | --- | --- | --- |
| NCT05306457 | Cedars-Sinai Medical Center | OTHER | HOSPITAL |
| NCT02611921 | Children's Hospital Medical Center, Cincinnati | OTHER | HOSPITAL |
| NCT01320982 | Sheba Medical Center | OTHER_GOV | HOSPITAL |

## 3. Characterization of Phase Not Applicable Studies

We manually reviewed a random sample of 20 trials classified as “Phase Not Applicable” on ClinicalTrials.gov. For each trial, we examined the brief title, official title, and study summary to identify the intervention type and primary study purpose. Trials were then grouped into categories such as nutritional interventions, approved-product or treatment-optimization studies, device or imaging studies, investigational therapeutic studies, mechanistic studies, observational studies, and post-approval monitoring studies. We also assessed whether the Phase Not Applicable designation appeared justified or whether a conventional phase may have been more appropriate.

**Supplementary Table 5**: Study types identified among trials classified as Phase Not Applicable.

| Phase N/A study category | n | % | Representative NCT examples |
| --- | --- | --- | --- |
| Clinical food, dietary, or nutritional interventions | 5 | 25% | NCT03797131; NCT00730184; NCT01746303 |
| Approved-product, drug, or procedure optimization and comparative-use studies | 4 | 20% | NCT01716767; NCT00792584; NCT03802435 |
| Device, physical-intervention, or diagnostic-imaging studies | 4 | 20% | NCT02891512; NCT05102045; NCT03757507 |
| Investigational therapeutic efficacy or drug-repurposing studies | 4 | 20% | NCT01193660; NCT05284175; NCT02221570 |
| Mechanistic or biomarker studies | 1 | 5% | NCT00538070 |
| Observational or disease-characterization studies | 1 | 5% | NCT00818103 |
| Post-approval monitoring or real-world treatment studies | 1 | 5% | NCT04159987 |
| **Total** | **20** | **100%** | — |

**Supplementary Table 6**: Review of Phase Not Applicable classifications in the sampled trials.

| Assessment | n | % | Representative NCT examples |
| --- | --- | --- | --- |
| Justified | 11 | 55% | NCT03797131; NCT05102045; NCT03757507 |
| Likely justified | 2 | 10% | NCT00538070; NCT01716767 |
| Uncertain; a numerical phase may have been appropriate | 3 | 15% | NCT00792584; NCT03802435; NCT02012335 |
| Likely not justified; a numerical phase should probably have been reported | 4 | 20% | NCT01193660; NCT05284175; NCT02221570 |
| **Total** | **20** | **100%** | — |

## 4. Trial selection flow diagram

**Supplementary Figure 1**: Flow diagram of the trial selection process.

## 5. Overview clinical trial purpose and status

**Supplementary Figure 2**: Clinical trial purpose and status

**A:** Clinical trials by primary purpose. **B:** Clinical trials by current status.

## 6. Clinical trials distribution over countries

**Supplementary Figure 3**: Heat map presenting number of clinical trials in neuroscience, per country registered in ClinicalTrials.gov.

## 7. Overview clinical trials sponsorship over phase and collaboration pairs

**Supplementary Figure 4**: Distribution of clinical trial lead sponsorship across phases (**A**) and collaboration pairs **(B**).

## 8. Comparison of phase 2 to phase 3 trials

**Supplementary Figure 5**: Comparison of phase 2 and phase 3 trials.

**A:** Percentage of trials by phase funded by Industry or University. **B:** Number of trials started per year funded by Industry or University. **C:** Percentage of trials by allocation type and phase. **D:** Percentage of trials by masking type and phase. **E:** Percentage of results reporting per phase over trial completion year.

## 9. Relationship between comparator category and masking type

**Supplementary Figure 6**: Comparator categories by masking type. Categories were derived from reported non-experimental arm types. Trials lacking design group data were labeled “No design group information”, and trials without a recorded non-experimental arm were labeled “No comparator information”. When a trial had multiple non-experimental arm types, these were combined; for example, trials with both active and placebo comparator arms were labeled “Active Comparator + Placebo Comparator.” The five most frequent categories are shown separately; all remaining combinations were grouped as “Other comparator combinations.” Band widths represent trial counts.

## 10.  Overview masking patterns by masking type

**Supplementary Figure 7**: Heatmap showing the frequency of specified masked group combinations across masking types.

## 11. Overview clinical trials outcomes

**Supplementary Figure 8**: Number of primary and secondary outcomes across trials

**A:** Distribution of primary outcomes in clinical trials. **B:** Distribution of secondary outcomes in clinical trials. Clinical trial size in terms of number of involved facilities and number of enrolled/anticipated participants. **C:** Number of facilities participating in a study. **D:** Number of enrolled/anticipated study participants.

**Supplementary Figure 9**: Top 20 conditions and drugs for phase 2 versus phase 3.

**A**: Phase 2 conditions. **B**: Phase 3 conditions. **C**: Phase 2 drugs. **D**: Phase 3 drugs.

## 12. Overview clinical trials in Parkinsonism cluster

**Supplementary Figure 10**: Comparison of the two identified Parkinsonism clusters: mentioned conditions and drug names in the right cluster (**A, B**) and left cluster (**C, D**).

**Supplementary Figure 11**: Comparison of the two identified Parkinsonism clusters: mentioned conditions and drug names in the right cluster (**A, B**) and left cluster (**C, D**).

## 13.  Construction of the ICD-11 and MeSH vocabulary

We constructed the neuropsychiatric disease vocabulary from the 2024 releases of ICD-11 and MeSH.

For MeSH, we selected 16 neurological hierarchy branches from the Nervous System Diseases category (C10): Autoimmune Diseases of the Nervous System (C10.114), Autonomic Nervous System Diseases (C10.177), Central Nervous System Diseases (C10.228), Chronobiology Disorders (C10.281), Cranial Nerve Diseases (C10.292), Demyelinating Diseases (C10.314), Nervous System Malformations (C10.500), Nervous System Neoplasms (C10.551), Neurocutaneous Syndromes (C10.562), Neurodegenerative Diseases (C10.574), Neurologic Manifestations (C10.597), Neuromuscular Diseases (C10.668), Neurotoxicity Syndromes (C10.720), Restless Legs Syndrome (C10.803), Sleep Wake Disorders (C10.886), and Trauma, Nervous System (C10.900). We parsed the 2024 MeSH descriptor XML file and retained descriptors associated with these selected hierarchy nodes. For each retained descriptor, we extracted the preferred name, MeSH identifier, tree number, disease class, and listed synonyms.

For ICD-11, we used the WHO ICD API to retrieve all English-language concepts under two parent chapters: *Diseases of the nervous system* and *Mental, behavioural or neurodevelopmental disorders*. All descendant concepts were recursively extracted, while retaining their parent–child relationships and broad disease-class assignments.

The two sources were then combined into a single terminology. MeSH preferred names and synonyms and ICD-11 concept titles were converted into individual term-level records. Exact duplicate terms were consolidated, while source-vocabulary and disease-class information were retained.

The code used to construct the terminology is available in the associated repository:
<https://github.com/Ineichen-Group/NeuroTrialNER/tree/main/data/neuro_diseases_terminology>
